# Smart stability indicating spectrophotometric methods for determination of modafinil: the promising treatment for post-covid neurological syndrome

**DOI:** 10.1186/s13065-022-00869-z

**Published:** 2022-10-21

**Authors:** Soha G. Elsheikh, Sally S. El-Mosallamy, Yasmin M. Fayez, Abeer M. E. Hassan

**Affiliations:** 1 Analytical Chemistry Department, Faculty of Pharmacy, October 6 University, Giza, Egypt; 2 Analytical Chemistry Department, Faculty of Pharmacy, Cairo University, Kasr El Aini, Cairo, 11562 Egypt

**Keywords:** Modafinil, Ratio difference, Derivative ratio, Mean centering, Ratio subtraction

## Abstract

Modafinil (MDF) is one of the neurostimulants with a potential effect in the COVID-19 ICU ventilated patients and post-COVID neurological syndrome treatment. Four rapid, simple and cost-effective stability indicating spectrophotometric methods were used for estimation of MDF in the presence of its acidic degradation product, namely; ratio difference (RD), first derivative of the ratio spectra (^1^DD), mean centering (MCR) and ratio subtraction method (RS). These methods were validated according to ICH guidelines and all methods revealed a good linearity in concentration range of (5-30 µg/mL) in addition to a good accuracy and precision with mean percentage recovery of 99.97 ± 0.305 for (RD), 100.10 ± 0.560 for (^1^DD), 100.02 ± 0.483 for (MCR) & 99.18 ± 1.145 for (RS) method. Specificity of the proposed methods was assessed and MDF was determined in the presence of up to 80% of its acidic degradation product for RD, ^1^DD, MCR and RS methods. The proposed methods were successfully applied for the determination of MDF in bulk powder and its tablet dosage form with mean percentage recovery of 100.33 ± 0.915 for (RD), 100.62 ± 0.985 for (^1^DD), 99.70 ± 0.379 for (MCR) and 100.21 ± 0.313 for (RS) method. The results obtained were statistically compared with those of official HPLC method and showed no significant difference with relevance accuracy and precision.

## Introduction

**Fig. 1:**
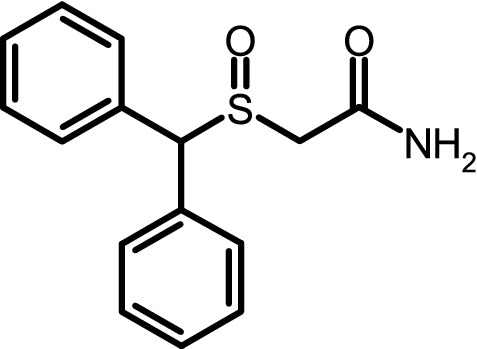
Chemical structure of Modafinil

Modafinil is 2- (benzhydryl sulfinyl) acetamide (Fig. 1) [1]. used as wakefulness promoting agent for its anti-oxidative and neuroprotective influences with unknown mechanism till now and it still controversial, it can be taken concomitantly with flecainide to treat narcolepsy [2], some clinical trials proof that MDF may relieve fatigue and neurobehavioral dysfunction in primary brain tumors patients [3].

COVID-19 patients who are mechanically ventilated may require greater doses of opioids and sedatives to avoid self-extubation and reduce ventilator-induced lung damage. MDF safety profile as well as its effect when administered to those hypoactive and lethargic critically patients make it an optimum choice in ventilated COVID-19 patients. MDF importance comes from its ability to avoiding tracheostomy. According to a recent literature study, tracheostomy patient with COVID-19 was completely extubated after receiving modafinil [4, 5].

Various analytical methods have been reported to estimate MDF individually and in concomitant with other drug in fluids or pharmaceutical dosage form using direct spectrophotometric method (Zero order) at 252 nm, first order derivative method at 234 nm [6], Direct spectrophotometric method at 260 nm [7], Colorimetric method using methyl orange at 525 nm and methylene blue at 664 nm [8], Colorimetric method utilizing 2,4-dinitrophenol and 1,2-naphthoquinone-4-sulphonate at wavelengths of 475 nm and 430 nm respectively [9], RP- HPLC [10, 11], capillary electrophoresis [12] and LC/MS [13] but few of them have been developed to estimate MDF in the presence of its degradation products using HPLC [14, 15], HPTLC [16] and capillary zone electrophoresis [17].

The stability of MDF was studied in few papers [14–18]. No spectrophotometric methods have been reported for the determination of MDF in the presence of its degradation product. So the purpose of this study is to develop simple, time saving, economic, precise and accurate stability-indicating spectrophotometric methods for determination of MDF in the presence of its degradation products.

## Experimental

### Apparatus and software

Shimadzu 1800 UVPC spectrophotometer using 1.00 cm quartz cells (Shimadzu, Kyoto, Japan). JENWAY hot plate, JENWAY 3510 pH meter (Stone, Staffs, UK), and MATLAB® software.

### Materials and reagents

#### Pure sample

Modafinil was kindly supplied by Mash Premiere Pharmaceutical Industries (Cairo, Egypt); its purity was found to be 100.03 ± 0.598 according to the official method [1].

#### Pharmaceutical dosage form

BRAVAMAX^®^ tablets, Batch No. 181142A and labeled to contain 200 mg/tab., manufactured by Chemipharm Pharmaceutical Industries (6th October, Egypt) were purchased from local market.

#### Degraded sample

Degradation product was prepared by refluxing 25 mg of modafinil powder with 5 N methanolic HCl for 12 h. at 80 °C, then the solution was neutralized with KOH, followed by purification to obtain pure powder of the degradation product which was checked by thin layer chromatography. as detailed in our previous work [18].

### Chemicals and reagents

All chemicals used throughout this study were of analytical grade, Methanol (Merck, Germany), Distilled water prepared-in house by Aquatron water still A8000 system, Hydrochloric acid 37% (Honeywell, USA), and Potassium Hydroxide (El NASR Pharmaceutical Chemicals Co., Abu-Zabaal, Cairo, Egypt).

### Solutions

#### Stock standard solution

Stock standard solutions of MDF and its degradation product (1 mg/mL) were prepared in methanol.

#### Working standard solutions

Working standard solutions of MDF and its degradation products (100 µg/mL) were prepared from their corresponding stock standard solutions in methanol.

### Procedure

#### Construction of calibration curves

Aliquots equivalent to 50–300 μg of MDF were accurately transferred from their working standard solutions into a set of 10 mL volumetric flasks and the volumes were completed using methanol then Zero order absorption spectra were scanned at (200–400 nm) and stored in the computer.

#### Ratio difference method (RD)

Ratio spectra were obtained by dividing the stored zero order absorption spectra of (5–30 µg/mL) MDF by the spectrum of its acidic degradation product (70 µg/mL) and stored in computer. The difference in the amplitudes between 224.4 nm and 232.8 nm was measured, Calibration graph was constructed and the linear regression equation between the difference in amplitude and concentration was computed for the determination of MDF.

#### First derivative of the ratio spectra (^1^DD)

The first derivative of the stored ratio spectra of MDF (5–30 µg/mL) were obtained using scaling factor 10 and Δλ = 4. The peak amplitude of the obtained spectra was measured at 241.8 nm. Calibration graph was plotted between peak amplitude and concentration, and then regression equation was calculated.

#### Mean centering (MCR)

The mean centering of the stored ratio spectra of MDF (5–30 µg/mL) were obtained using MATLAB^®^ software and calibration graph of the mean centered values at 224 nm was constructed against their corresponding concentrations and regression equation was calculated.

#### Ratio subtraction method (RS)

Ratio subtraction method was manipulated, and the stored zero order absorption spectra of MDF (5–30 µg/mL) manipulated to its second derivative spectra, calibration curve was constructed by plotting absorbance at 223.7 nm of second derivative spectra of MDF against the corresponding concentration and then linear regression equation was calculated.

#### Analysis of laboratory prepared mixtures

Laboratory prepared mixtures containing different percentages (20–80%) of the degradation product were analyzed. The procedures under construction of calibration curves for RD, ^1^DD and MCR were followed. Ratio subtraction method was realized via working on laboratory prepared mixtures and four steps were applied. The first step was performed by dividing synthetic lab mixtures on suitable divisor (70 µg/mL of the acidic degradation product); the second step was subtracting the constant value (plateau region) from the obtained ratio spectra. The third step was multiplying the obtained spectra by the spectrum of the divisor to obtain the original spectra of MDF, and the final step was manipulating the spectra of the previous step to obtain second derivative spectra. MDF concentrations were calculated from the regression equation of each method.

#### Application of pharmaceutical dosage preparation

Ten tablets of BRAVAMAX^®^ were weighed and grinded well, a portion of the powder equivalent to 50 mg MDF was weighed and transferred to 50-mL volumetric flask, 30 mL of methanol was added and sonicated for 25 min and the solution was filtrated into another 50-mL volumetric flask. The volume completed to the mark with the same solvent to prepare stock tablet solution equivalent to (1 mg/mL) MDF. Ten milliliters from (1 mg/mL) MDF transferred into 100 mL volumetric flask using the same solvent to complete volume to get working solution of (100 µg/mL). Then the procedures completed as detailed under each method.

## Results and discussion

Stability testing assesses for pharmaceutical products to ensure their safety and effectiveness through subjecting them to normal and accelerated conditions which help in the determination of shelf life, storage condition, ensure quality and suitable packaging material before supplying to market [19].

In our previous work degradation of MDF was studied via series of trials with different degradation conditions until prove its liability to acid and base. In addition to confirming that no oxidative degradation was observed upon using different oxidative degradation conditions. Structure elucidation was performed using IR & mass spectrometry then degradation pathway was predicted[18].

Spectrophotometry is an economical and simple method in comparison with HPLC method besides its availability in all quality control laboratories. The main problem facing the analysts is the overlapping spectra of the drug and its degradation products. Manipulating ratio spectra was the excellent solution to face this obstacle [20].

### RD method

**Fig. 2:**
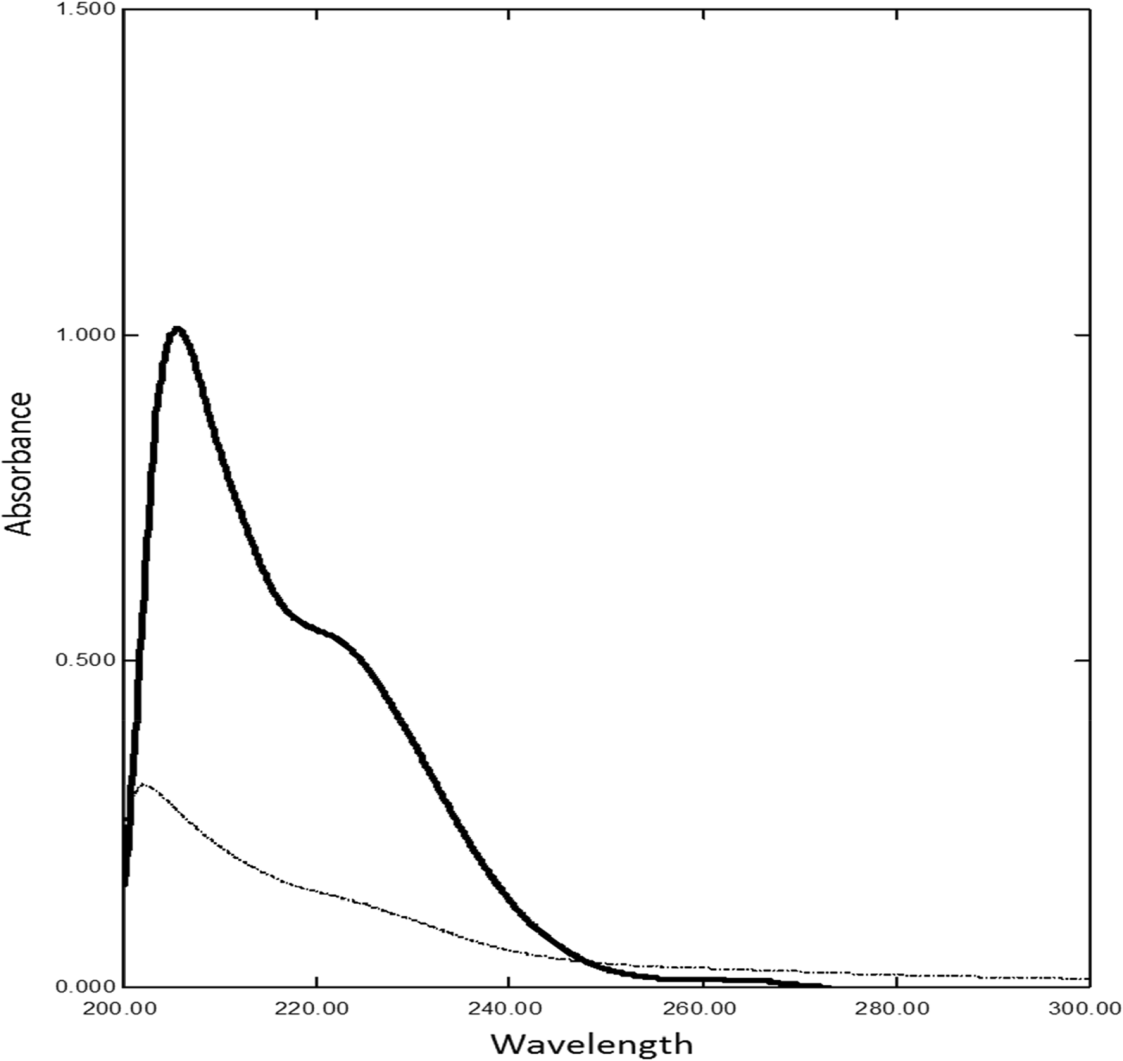
Zero order absorption spectra of (10 µg/mL) modafinil (–) and (70 µg/mL) of its acidic degradation (**-—-**) in methanol

Absorption spectra of MDF and its degradation product show severe overlap (Fig. 2), which hinders the determination of MDF using direct spectrophotometric method. In addition, classical derivative spectrophotometric method failed in determination of MDF. So, we resorted to different spectrophotometric methods manipulating ratio spectra.

**Fig. 3:**
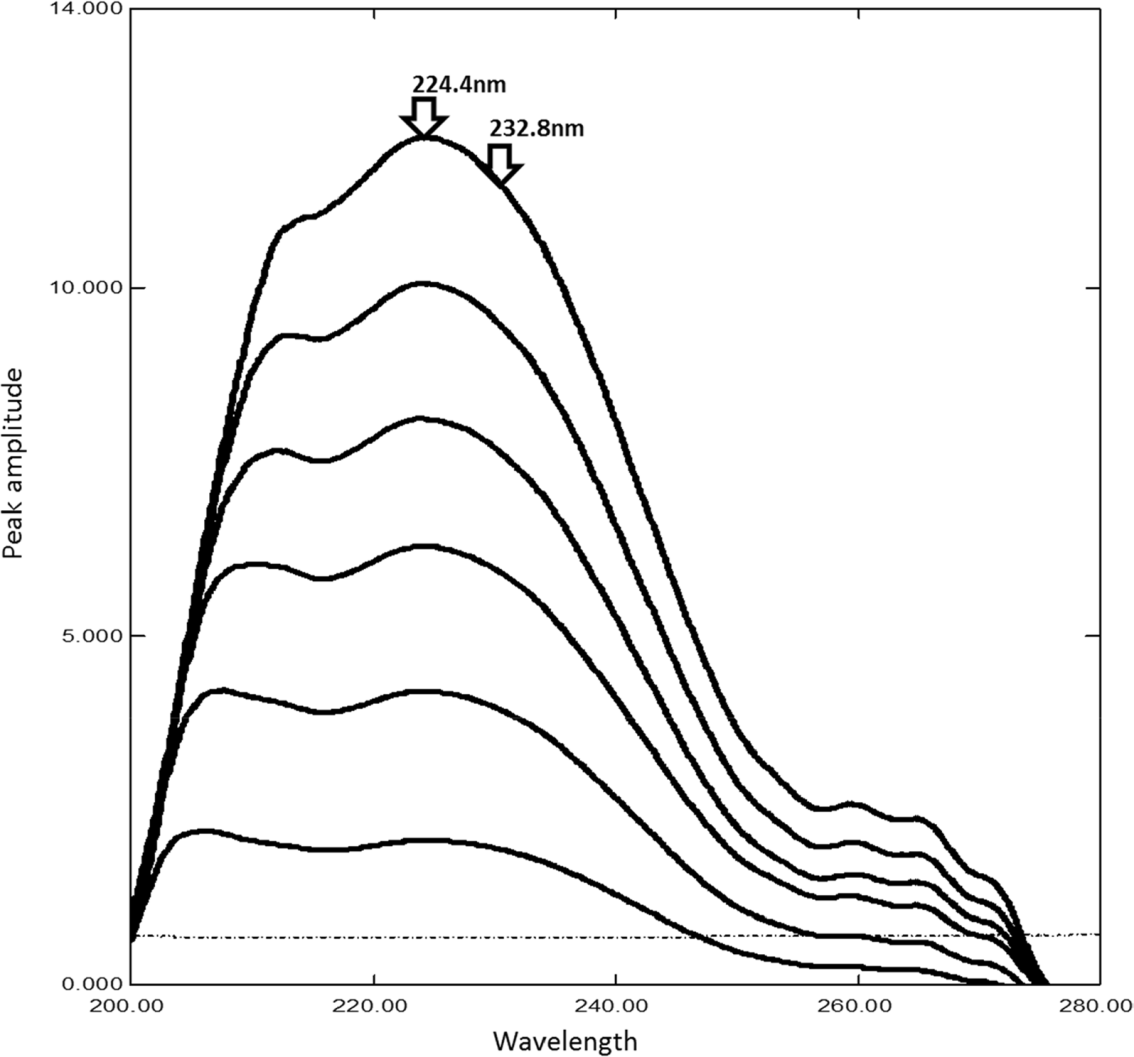
Ratio spectra of modafinil 5-30 µg/mL(–) and 50 µg/mL of its acidic degradation product (**-—-**) using 70 µg/mL of degradation as a divisor in methanol

Simplicity, accuracy and reproducibility are the most remarkable features of ratio difference method [21, 22], which involves two main requirements [23], the first one is the divisor selection which successfully chosen to be 70 µg/mL of the acidic degradation product that was showing minimal noise. The second requirement is the selection of two wavelengths which found to be λ_1_ (224.4 nm) and λ_2_ (232.8 nm) (Fig. 3). A calibration curve was constructed representing the relationship between the ΔP of the selected wavelengths and the corresponding concentrations. Good linearity, maximum sensitivity with a satisfactory recovery in the range of (5–30 µg/mL) of the drug were obtained as listed in (Table 1). MDF could be determined in the presence of up to 70% of its degradation product.

**Table 1:** Assay validation sheet of the proposed methods for the determination of pure samples of modafinil

Parameter	RD	^1^DD	MCR	RS
Accuracy (mean ± SD)	99.97 ± 0.305	100.10 ± 0.560	100.02 ± 0.483	99.18 ± 0.1.145
Precision (RSD %)				
Repeatability^*^	0.764	1.104	1.008	0.939
Intermediate precision^**^	0.855	0.958	0.952	1.112
Linearity				
Slope	0.0362	0.1525	0.1812	0.0032
Intercept	0.1292	0.2614	0.235	0.0002
Correlation coefficient (r)	0.9999	0.9999	0.9999	0.9998
Range	(5-30 µg/mL)	(5-30 µg/mL)	(5-30 µg/mL)	(5-30 µg/mL)
Limit of quantification (µg/mL)	0.707	0.877	1.094	1.389
Limit of detection (μg/mL)	0.233	0.290	0.36	0.458
Robustness (RSD %)^***^				
[chosen wavelength ± 2 nm]	1.737	0.689	0.779	1.103

^*^The intra-day (n = 3), RSD of three concentration 20, 25, 30 µg/mL of MDF triplicate analysis within the day and

^**^The inter-day (n = 3), RSD of three concentration 20, 25, 30 µg/mL of MDF triplicate analysis/day on three successive days by the suggested spectrophotometric methods

^***^RSD of MDF concentration of 15 µg/mL -repeatedly 3 times- were determined under minor wavelength variation [chosen wavelength for each method ± 2 nm]

### ^1^DD method

**Fig. 4:**
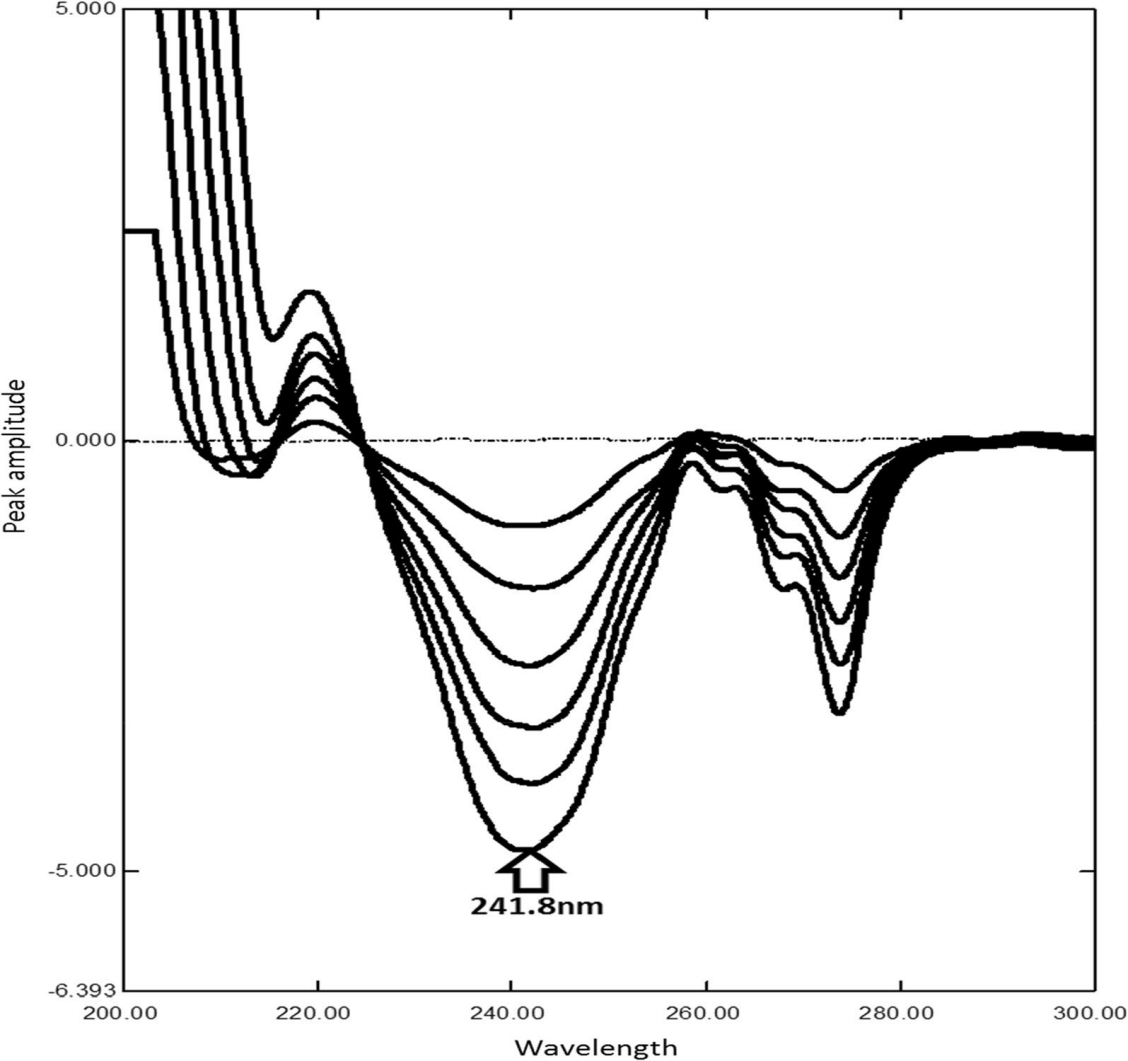
First derivative of ratio spectra of 5-30 µg/mL modafinil (–) and 50 µg/mL of degradation product (**—**) using 70 µg/mL of degradation as a divisor at (241.8 nm) in methanol

First derivative of the ratio spectra method is one of the simplest spectrophotometric method [21, 22]. ^1^DD easily applicable for the quantitative analysis of MDF in the presence of its acidic degradation despite of the overlapping spectra of them. It involves the conversion of zero order spectra to ratio spectra using (70 µg/mL) of MDF acidic degradation product as divisor, then first derivative spectra were obtained using scaling factor 10 and Δλ = 4 nm as in (Fig. 4). Calibration curve was constructed representing the relationship between peak amplitude at 241.8 nm and the corresponding concentrations. Regression equation was calculated in the range of (5–30 µg/mL) as listed in (Table 1). MDF could be determined in the presence of up to 80% of its degradation product.

### Mean centering

**Fig. 5:**
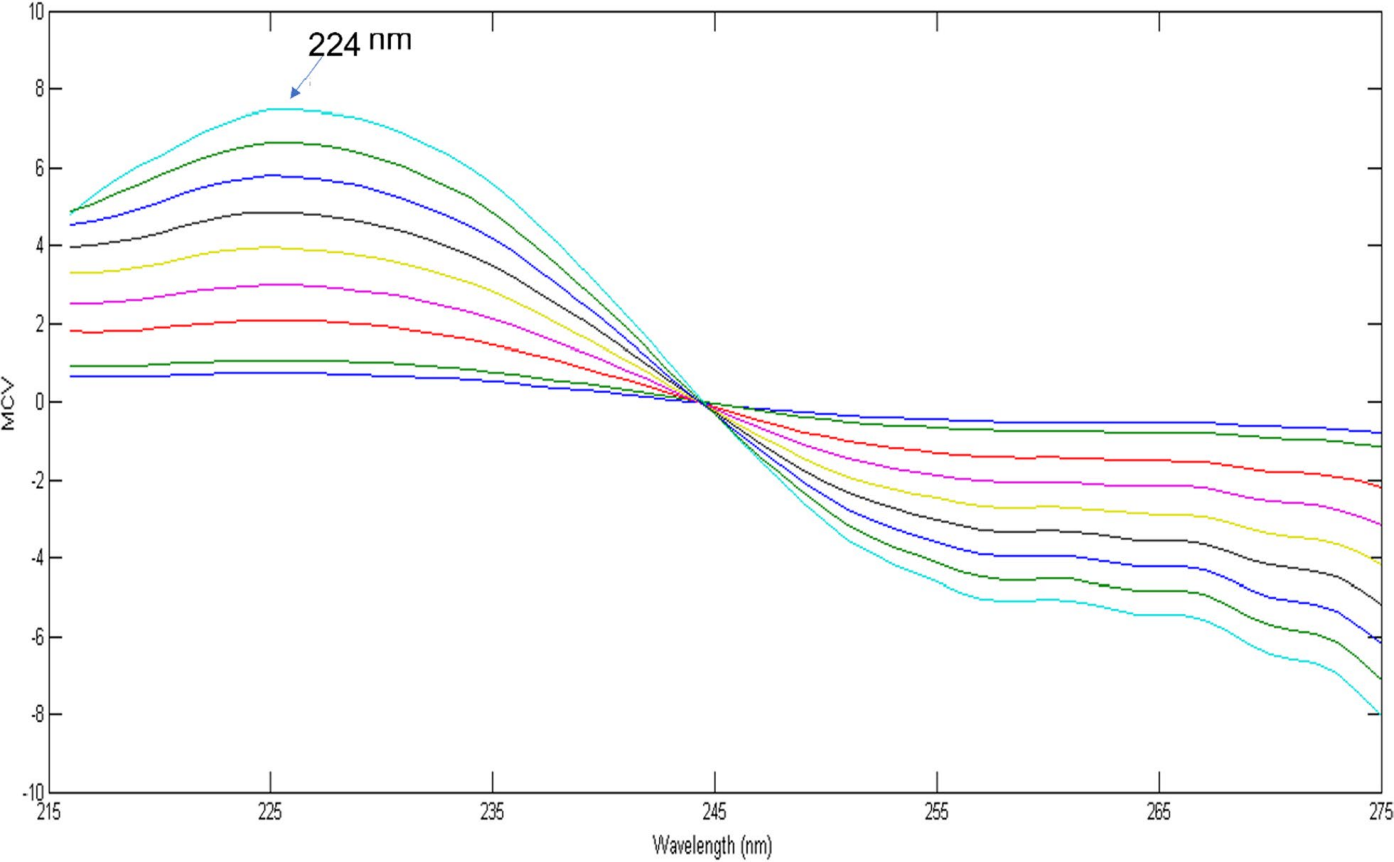
Mean centering of ratio spectra of MDF 5-30 µg/mL using the spectrum of 70 µg/mL of degradation as a divisor at 226 nm in methanol

Calibration graph was constructed in the range of MDF of (5–30 µg/mL) for the values of the mean centered spectra at 224 nm, after transferring the ratio spectra to MATLAB® for subsequent manipulation. As this method based on mean centering of ratio spectra rather than the derivative steps, therefore signal-to-noise ratio is enhanced [22, 24, 25] (Fig. 5). Regression equation was computed (Table1). MDF could be determined in the presence of up to 80% of its degradation product.

### Ratio subtraction method

**Fig. 6:**
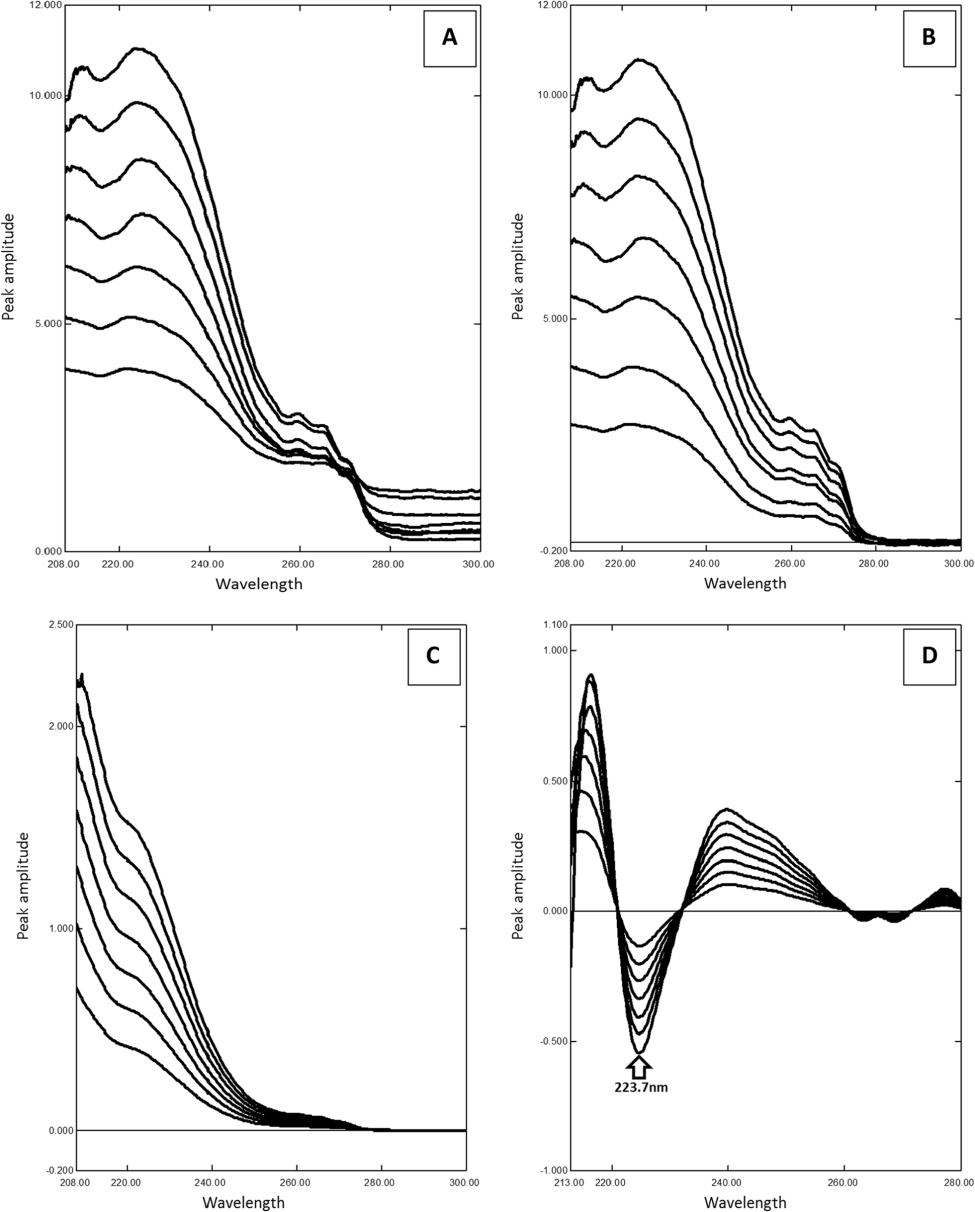
**A** Ratio spectra of laboratory prepared mixtures of modafinil and its degradation product using 70 µg/mL of its degradation product as a divisor. **B** The obtained ratio spectra after subtraction of the constant. **C** The obtained spectra of modafinil after multiplication by the divisor. **D** Second derivative of the obtained spectra of the laboratory prepared mixtures

Ratio subtraction method [21, 26] is implied as degradation product spectrum is more extended than the absorption spectrum of MDF (Fig. 2). This method was achieved through dividing laboratory prepared mixtures by a carefully chosen divisor 70 µg/mL acid degradation thus leading to a straight line parallel to zero line at range 275 nm- 333 nm referring to constant (Fig. 6A). After that subtracting this constant from the obtained spectra (Fig. 6B). The obtained spectrum was then multiplied by the divisor spectrum (Fig. 6C). Finally, the original spectrum of MDF was obtained and manipulated to its second derivative spectrum, MDF can be determined at 223.7 nm (Fig. 6D). It was difficult to construct the calibration curve from zero order spectra because of two main reasons; first, MDF main peak was showed in zero spectra at range (204-211 nm), while the second reason was the peak shifting in this range as the concentration increased. A first derivative spectrum was also hindered to be constructed because of the peak shifting. The linear correlation was constructed in the range of (5–30 µg/mL) (Table 1). MDF could be determined in the presence of up to 80% of its degradation product.

### Method validation

ICH guidelines for method validation [27] were followed for all the proposed methods. Results of accuracy, precision, and repeatability are presented in (Table 1). Robustness was realized by determining the sample under small wavelength variation about ± 2 nm from the specified wavelength as shown in (Table 1).

### Application of the proposed methods in assay of laboratory prepared mixtures

Specificity of the proposed methods was assessed by the analysis of different laboratory prepared mixtures containing different percentages (20–80%) of MDF standard solution and the degradation product. The recovery percentage and RSD% were acceptable enough to assess the specificity as shown in (Table 2) [28].

**Table 2:** Determination of modafinil in laboratory-prepared mixtures by the proposed spectrophotometric methods

Degradation Product %	RD	^1^DD	MCR	RS
20%	99.07	100.38	101.47	100.37
30%	100.45	100.72	100.66	98.96
40%	98.36	100.39	100.79	100.00
50%	99.82	100.64	99.51	98.25
60%	99.12	99.21	99.73	98.55
70%	100.56	100.29	101.76	99.23
80%	104.76*	101.08	98.97	98.56
Mean	99.57	100.39	100.41	99.13
SD	0.864	0.586	1.041	0.792
RSD%	0.868	0.583	1.037	0.799

^*^Rejected values according to rejection rule [28]

### Application of the proposed methods in assay of pharmaceutical formulation

The suggested methods were effectively used for the analysis of MDF in Bravamax^®^ tablets and the validity of the methods was further assessed by applying standard addition technique (Table 3).

**Table 3:** Quantitative determination of modafinil in Bravamax^®^ tablet by the suggested methods and results of application of standard addition technique

BRAVAMAX^®^ tablets 200 mg Batch No. 181142A			RD	^1^DD	MCR	RS
Found%^*^ ± SD%			100.33 ± 0.915	100.62 ± 0.985	99.70 ± 0.379	100.21 ± 0.313
Standard Addition Technique	Taken (µg/mL)	Added(µg/mL)	Recovery %			
	10	5	101.04	99.17	100.42	102.08
		10	101.27	100.72	100.13	101.88
		15	100.48	100.25	100.43	100.25
	Mean ± SD%		100.93 ± 0.410	100.05 ± 0.793	100.32 ± 0.171	101.40 ± 1.004

^*^average of three determination

No considerable difference was observed in the statistical comparison between the results obtained from the proposed methods of the pure drug samples and those results from the official method [1] (Table 4)

**Table 4:** Statistical analysis of the results obtained by the proposed methods and the reported method for the determination of modafinil in pure powder form

	RD	^1^DD	MCR	RS	Reported method*
Mean	99.97	100.10	100.02	99.18	100.03
SD	0.305	0.560	0.483	1.145	0.598
Variance	0.093	0.313	0.233	1.311	0.358
n	6	6	6	6	7
Student’s t-test**	0.221 (2.20)	0.217 (2.20)	0.033 (2.20)	1.631 (2.20)	
F value**	3.85 (4.95)	1.14 (4.95)	1.54 (4.95)	3.66 (4.39)	

^*^HPLC method using C18 column, acetonitrile: 25 mM phosphate buffer (35:65 v/v) as a mobile phase and UV detection at 220 nm [1]

^**^Figures between parentheses represent the corresponding tabulated values of t and F at *p* = 0.05

## Conclusion

Main reason for currently underway studies on modafinil in the last 3 years is to prioritize its effectiveness in the post neurological syndrome caused by global epidemic [COVID-19]. Four simple, accurate and time-saving stability indicating spectrophotometric methods were developed for quantitative estimation of MDF in the presence of its degradation products. First derivative of the ratio spectra, MCR and RS methods resolve the challenging interference of MDF spectra in the presence of 80% of its degradation products. To the best of our knowledge no spectrophotometric methods has been published for estimation of MDF in a presence of its degradation products.

Taking into consideration, the easiest manipulation of RD method (a one-step process). that achieves good accuracy and high reproducibility While ^1^DD and MCR both are two-step methods but MCR provides enhanced signal-to-noise ratio as it is based on mean centering of ratio spectra rather than the derivative steps which enhance the selectivity. Ratio subtraction method has the advantage of restoring the original spectra of proposed drug despite being limited for analyzing binary mixtures where the spectrum of one component must extend over the other. Therefore, Application of the suggested methods convenient to MDF quality control routine analysis, because of their simplicity and validity according to ICH guideline.

## Data Availability

Spectrophotometric data obtained from spectrophotometer software. Datasets generated and/or analyzed during the current study are available from the corresponding author on reasonable request.

## Abbreviations

MDF: Modafinil
RD: Ratio difference
^1^DD: First derivative of the ratio spectra
MCR: Mean centering
RS: Ratio subtraction method
HPLC: High performance liquid chromatography
RP-HPLC: Reversed phase High performance liquid chromatography
LC/MS: Liquid chromatography—mass spectrometry
HPTLC: High performance thin—layer chromatography
IR: Infrared spectrophotometer
LC/MS/MS: Ultra-performance liquid chromatography mass spectrophotometer
tab: Tablet
μg: Microgram
mL: Milliliter
nm: Nanometer
ICH guidelines: International council on harmonization
SD: Standard deviation
RSD: Relative standard deviation
QC: Quality control
ICU: Intensive care unit
